# Ultrasonography in Detection of Renal Calculi in Children; a Systematic Review and Meta-analysis

**Published:** 2019-11-24

**Authors:** Mojtaba Fazel, Mohammed I M Gubari, Mahmoud Yousefifard, Mostafa Hosseini

**Affiliations:** 1 Pediatric Chronic Kidney Disease Research Center, Tehran University ofMedical Sciences, Tehran, Iran.; 2 Department of Pediatrics, Valiasr Hospital, Imam KhomeiniMedical Complex, Tehran University ofMedical Science, Tehran.; 3 CommunityMedicine, College ofMedicine, University of Sulaimani, Sulaimani, Iraq.; 4 Physiology Research Center, Iran University ofMedical Sciences, Tehran Iran.; 5 Department of Epidemiology and Biostatistics, School of Public Health, Tehran University of Medical Sciences, Tehran, Iran.

**Keywords:** Kidney calculi, ultrasonography, diagnostic imaging, pediatrics

## Abstract

**Introduction:**

Although numerous studies have been done to evaluate the diagnostic value of ultrasonography in diagnosis of renal calculi in children, there is still no consensus. Therefore, in the present systematic review and meta-analysis, we aimed to evaluate the diagnostic accuracy of ultrasonography in identifying renal stones in children.

**Methods:**

A comprehensive search of the electronic databases including Medline, Embase, Scopus and Web of Science was conducted up to July 2019. Diagnostic accuracy studies in children were included. Data was summarized and pooled. Area under the curve (AUC), sensitivity, specificity, diagnostic score and diagnostic odds ratio were reported with 95% confidence interval (95% CI).

**Results:**

Data from 7 articles were included. Pooled analysis showed that the area under the curve of ultrasonography in diagnosis of pediatric renal calculi was 0.94 (95% CI: 0.92 to 0.96). The sensitivity and specificity of this diagnostic modality were 0.80 (95% CI: 0.70 to 0.87) and 1.00 (95% CI: 0.84 to 1.00), respectively. Diagnostic score and diagnostic odds ratio of ultrasonography in detection of renal calculi were 110.32 (95% CI: 2.88 to 19.76) and 82362.41 (95% CI: 17.80 to 3.8 × 10^8^), respectively.

**Conclusion:**

Overall, the low level of evidence indicates that sensitivity and specificity of ultrasonography in detecting renal calculi in children are 80% and 100%, respectively. However, due to the serious limitations of the included studies, well-designed prospective diagnostic accuracy studies are recommended for future studies.

## Introduction:

Renal calculi are a common cause of hematuria as well as abdominal and flank pains. Statistics have shown a high incidence of this condition, with one in 10 people developing renal calculi. The prevalence of renal calculi in children has not been evaluated in population-based studies. However, local and hospital-based studies have shown an increase in reported cases of pediatric renal calculi. The prevalence of renal calculi in 1999 was about 18.4 children per 100,000 population, compared to 57 children per 100,000 population in 2008 (1). Although the prognosis of renal calculi in children is often good, mortality and persistent disability have also been reported in some cases. 

Although computed tomography (CT) scans are the most important diagnostic method for renal calculi in children, the risk of exposure to radiation, which is associated with the risk of cancer and its high medical costs, is a limitation of its use. However, there is no evidence that CT scans lead to improvement of patient outcomes (despite their high sensitivity). These limitations of CT indicate the need for alternative diagnostic methods, which should be reliable in addition to preventing exposure to radiation and being inexpensive. Ultrasonography may be a reliable alternative for this purpose (2-5).

High diagnostic speed and portability has made ultrasonography the first diagnostic step in many clinical conditions. However, the diagnostic accuracy of ultrasonography is highly dependent on the skill of the operator and is not very reliable in identifying parenchymal injuries and injuries that are free of bleeding or free fluid flow (6-8). Yet, technological advancements in ultrasound equipment in recent years have improved the quality of images, especially their spatial resolution. Consequently, with a brief training of physicians, the diagnostic sensitivity of the test can increase significantly. This has led to the use of ultrasonography in identifying various clinical conditions (9-11) and in some cases its performance is even better than other imaging modalities (9, 10, 12-14).

Although several studies have been done to evaluate the diagnostic value of ultrasonography in detection of renal calculi in children, there is still no consensus on this (15-17). Systematic reviews and meta-analyses are one of the solutions that can be used to provide a reliable conclusion. In recent years, some review articles have been conducted to evaluate the value of ultrasonography and CT in identifying renal calculi in children. However, none of them have meta-analytically evaluated the value of these modalities in identifying renal calculi (15-19). Therefore, in the present systematic review and meta-analysis, we aimed to evaluate the diagnostic accuracy of ultrasonography in identifying renal stones in children.

## Methods:

**Search strategy**

A comprehensive literature search of the electronic databases including Medline, Embase, Scopus and Web of Science was conducted up to July 2019. A search strategy based on keywords related to ultrasound and CT scan including “ultrasound” or “sonography” or “ultrasonography” or “computed tomography scan” or “CT scan” or “tomography scan” in combination with words related to renal calculi including “renal stone” or “kidney stone” or “kidney calculi” or “renal calculi” or “urolithiasis” was performed. Since the present meta-analysis focuses on child population, child-related keywords were also included in the search strategy. To find additional articles, manual search was carried out using the references of relevant studies. Keywords were selected as widely as possible so that no study would be omitted. Although only human studies were included in the present meta-analysis, the human studies related filter was not used. The keywords used were obtained using MeSH of PubMed database, Emtree section of Embase database and search in the title of related articles.

Furthermore, three strategies were employed for searching Gray literature. Firstly, the theses section of the ProQuest database was searched. Secondly, authors of related articles were contacted to obtain unpublished or in-print data. Finally, Google search engine and Google Scholar were used to obtain additional resources. Applying these strategies resulted in the inclusion of an additional article to the present meta-analysis. The query on the Medline database (via PubMed) is presented in appendix (Table S1).

**Table 1:** Summary of included studies

**Author**	**Sample size**	**Sampling design**	**Age (year)**	**NO. Boys**	**Golden standard**	**Probe**	**Operator**	**US stone criteria**	**US to CT interval (hours)**	**TP**	**TN**	**FP**	**FN**
Hu, 2010 (22)	1062	Prospective	1 to 10	565	CT urography	High frequency probe	Radiologist	NR	NR	36	1013	0	13
Johnson, 2011 (23)	42	Retrospective	3 to 25	24	CT	NR	NR	NR	NR	37	1	0	4
Palmer, 2005 (24)	54	Retrospective	3 to 18	28	CT	NR	NR	NR	NR	10	37	0	7
Passerotti, 2009 (25)	50	Prospective	2 to 18	NR	CT	Variable probe	Radiologist	AS	0.5 to 8	26	16	0	8
Roberson, 2018 (26)	69	Retrospective	5 to 19	35	CT	Variable probe	Pediatric sonographer	EF, TA, AS	0 to 24	20	38	1	10
Smith, 2000 (27)	28	Retrospective	1 to 13	20	Patient follow up	NR	NR	NR	NR	23	4	0	1
Verhagen, 2019 (28)	77	Retrospective	1 to 17	NR	CT	1.9 kHz	Radiologist	EF, AS, TA	NR	59	7	0	11

**Table 2:** Risk of bias of in included studies based on quality assessment of diagnostic accuracy studies 2 (QUADAS-2) recommendations

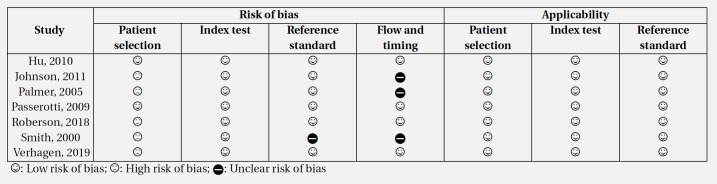

**Table 3:** Subgroup analysis for assessment of ultrasonography in detection of renal calculi

**Variable**	**Number of studies**	**Sensitivity (95% CI)**	**P**	**Specificity (95% CI)**	**P**
**Overall **	7	0.80 (0.70 to 0.87)	---	1.0 (0.84 to 1.0)	---
**Study design**					
Retrospective	5	0.80 (0.70 to 0.87)	---	1.0 (0.75 to 1.0)	---
Prospective	2	NA	---	NA	
**Operator**					
Radiologist/Pediatric sonographer	4	0.76 (0.68 to 0.83)	0.14	1.0 (0.86 to 1.0)	<0.0001
Not reported	3	0.85 (0.75 to 0.96)		1.0 (1.0 to 1.0)	
**Reporting of US criteria**					
No	4	0.82 (0.72 to 0.93)	0.09	1.0 (1.0 to 1.0)	<0.0001
Yes	3	0.77 (0.65 to 0.90)		0.99 (0.95 to 1.0)	
**Study year**					
Before 2010	4	0.78 (0.66 to 0.89)	0.33	1.0 (1.0 to 1.0)	<0.0001
After 2010	3	0.82 (0.72 to 0.89)		1.0 (0.93 to 1.0)	

**Figure 1:**
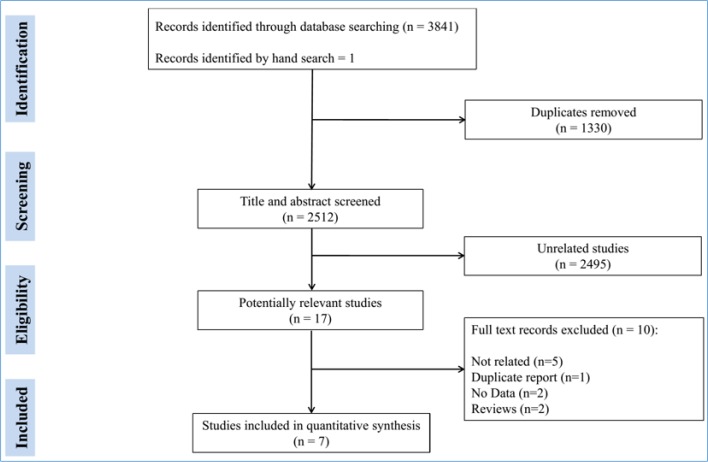
Preferred Reporting Items for Systematic Reviews and Meta-Analyses (PRISMA) flow diagram of the present meta-analysis

**Figure 2:**
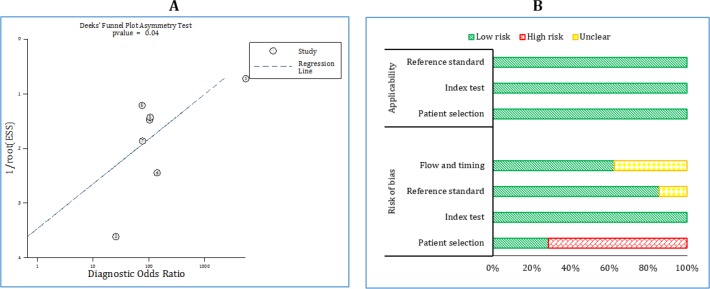
Publication bias (A) and risk of bias (B) among included studies.

**Figure 3:**
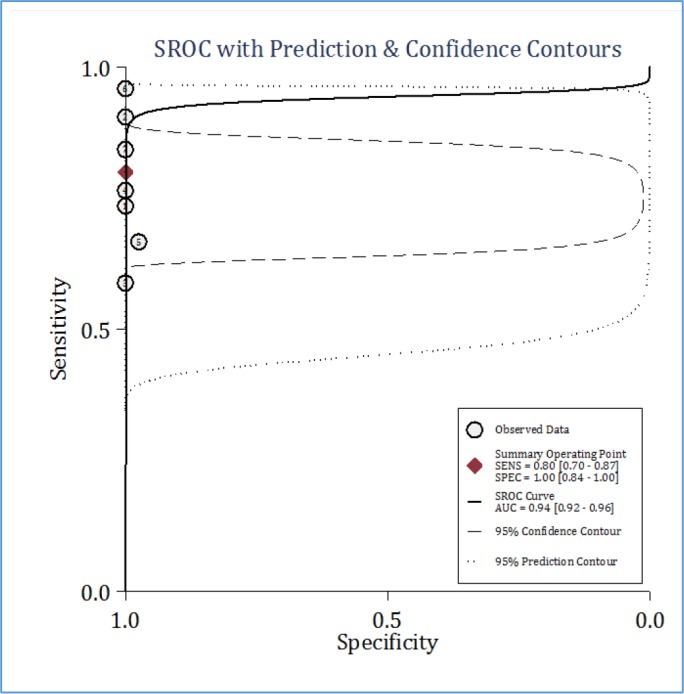
Summary receiver operating characteristic (SROC) curve of ultrasonography in detection of renal calculi in children. AUC: Area under the curve; SENS: Sensitivity; SPEC: Specificity

**Figure 4:**
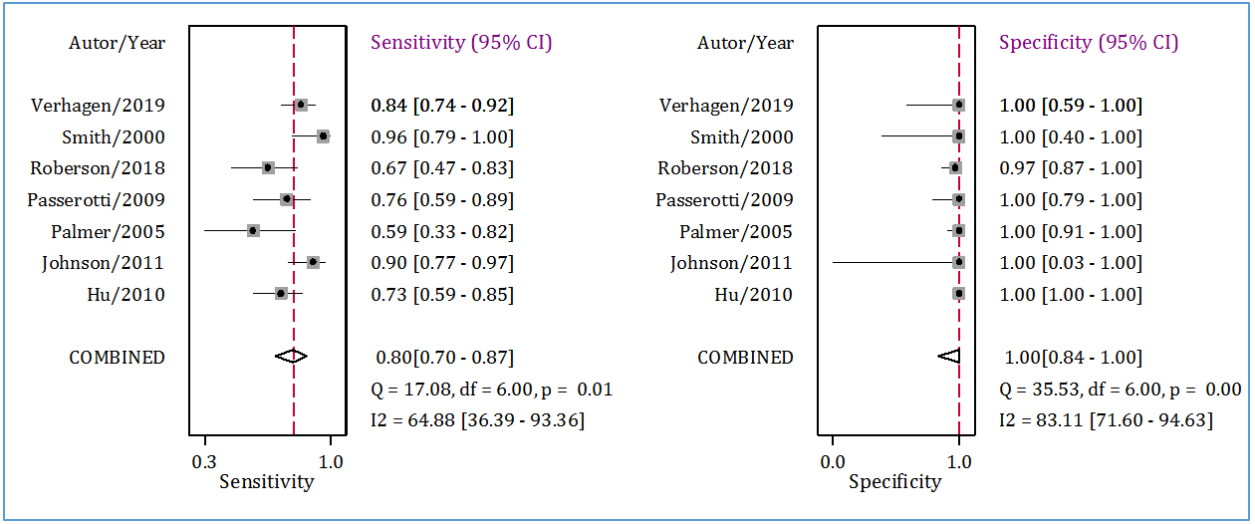
Sensitivity and Specificity of ultrasonography in detection of renal calculi in children. CI: Confidence Interval.

**Figure 5:**
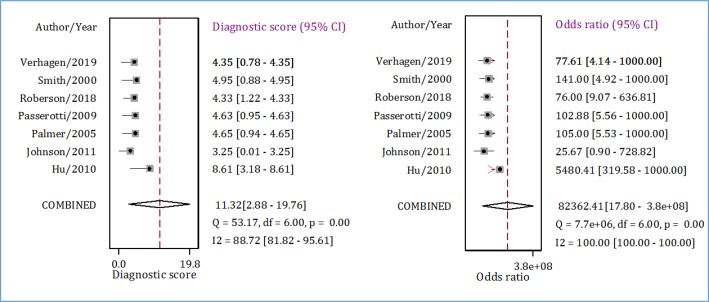
Diagnostic score and diagnostic odds ratio of ultrasonography in detection of renal calculi in children. CI: Confidence Interval.

**Table S1:** Search query of PubMed

"Ultrasonography"[mh] OR Ultrasonography[tiab] OR echography[tiab] OR Doptone[tiab] OR Duplex Echography[tiab] OR Echogram[tiab] OR Echoscopy[tiab] OR Echosound[tiab] OR High Resolution Echography[tiab] OR Scanning, Ultrasonic[tiab] OR Sonogram[tiab] OR Sonography[tiab] OR Ultrasonic Detection[tiab] OR Ultrasonic Diagnosis[tiab] OR Ultrasonic Echo[tiab] OR Ultrasonic Examination[tiab] OR Ultrasonic Scanning[tiab] OR Ultrasonic Scintillation[tiab] OR Ultrasonography[tiab] OR Ultrasound Diagnosis[tiab] OR Ultrasound Scanning[tiab] OR Ultrasound Imaging[tiab] OR Imaging, Ultrasound[tiab] OR Imagings, Ultrasound[tiab] OR Ultrasound Imagings[tiab] OR Ultrasonic Imaging[tiab] OR Imaging, Ultrasonic[tiab] OR Sonography, Medical[tiab] OR Medical Sonography[tiab] OR Diagnostic Ultrasound[tiab] OR Diagnostic Ultrasounds[tiab] OR Ultrasound, Diagnostic[tiab] OR Ultrasounds, Diagnostic[tiab] OR Echotomography[tiab] OR Diagnosis, Ultrasonic[tiab] OR Diagnoses, Ultrasonic[tiab] OR Ultrasonic Diagnoses[tiab] OR Ultrasonic Diagnosis[tiab] OR Echotomography, Computer[tiab] OR Computer Echotomography[tiab] OR Tomography, Ultrasonic[tiab] OR Ultrasonic Tomography[tiab] "Calculi"[mh] OR "Urinary Calculi"[mh] OR "Urolithiasis"[mh] OR "Ureterolithiasis"[mh] OR "Ureteral Calculi"[mh] OR "Kidney Calculi"[mh] OR "Urinary Bladder Calculi"[mh] OR Calculi[tiab] OR Urinary Calculi[tiab] OR Urolithiasis[tiab] OR Ureterolithiasis[tiab] OR Ureteral Calculi[tiab] OR Kidney Calculi[tiab] OR Urinary Bladder Calculi[tiab] OR Calculus[tiab] OR Urinary Stones[tiab] OR Calculi, Urinary[tiab] OR Calculus, Urinary[tiab] OR Urinary Calculus[tiab] OR Urinary Stones[tiab] OR Stone, Urinary[tiab] OR Stones, Urinary[tiab] OR Urinary Stone[tiab] OR Urinary Tract Stones[tiab] OR Stone, Urinary Tract[tiab] OR Stones, Urinary Tract[tiab] OR Urinary Tract Stone[tiab] OR Urinary Lithiasis[tiab] OR Lithiasis, Urinary[tiab] OR Ureterolithiases[tiab] OR Calculi, Ureteral[tiab] OR Calculus, Ureteral[tiab] OR Ureteral Calculus[tiab] OR Calculi, Kidney[tiab] OR Calculus, Kidney[tiab] OR Kidney Calculus[tiab] OR Nephrolith[tiab] OR Renal Calculus[tiab] OR Renal calculi[tiab] OR Kidney Stone[tiab] OR Stone, Kidney[tiab] OR Stones, Kidney[tiab] OR Renal Calculi[tiab] OR Calculi, Renal[tiab] OR Calculus, Renal[tiab] OR ladder Calculi, Urinary[tiab] OR Bladder Calculus, Urinary[tiab] OR Calculi, Urinary Bladder[tiab] OR Calculus, Urinary Bladder[tiab] OR Urinary Bladder Calculus[tiab] OR Bladder Stones[tiab] OR Bladder Stone[tiab] OR Stone, Bladder[tiab] OR Stones, Bladder[tiab] OR Calculi of Urinary Bladder[tiab] OR Urinary Bladder Stones[tiab] OR Bladder Stone, Urinary[tiab] OR Bladder Stones, Urinary[tiab] OR Stone, Urinary Bladder[tiab] OR Stones, Urinary Bladder[tiab] OR Urinary Bladder Stone[tiab] OR Vesical Calculi[tiab] OR Calculi, Vesical[tiab] OR Calculus, Vesical[tiab] OR Vesical Calculus[tiab] OR Bladder Calculi[tiab] OR Bladder Calculus[tiab] OR Calculi, Bladder[tiab] OR Calculus, Bladder[tiab] OR Cystoliths[tiab] OR Cystolith[tiab] OR Nephrolithiasis[tiab] OR Calculosis, Kidney[tiab] OR Calculus, Kidney[tiab] OR Familial Nephrolithiasis[tiab] OR Kidney Calculi[tiab] OR Kidney Calculosis[tiab] OR Kidney Calculus[tiab] OR Kidney Calix Stone[tiab] OR Kidney Calyx Stone[tiab] OR Kidney Lithiasis[tiab] OR Kidney Pelvis Stone[tiab] OR Kidney Stone[tiab] OR Kidney Stone Passage[tiab] OR Kidney Stone, Pelvis[tiab] OR Renal calculi[tiab] OR Nephrolith[tiab] OR Nephrolith Passage[tiab] OR Renal Calculus[tiab] OR Renal Lithiasis[tiab] OR Renal Pelvis Stone[tiab] OR Renal Stone[tiab] OR Renolithiasis[tiab] OR Stone, Kidney[tiab] OR Coral Stone[tiab] OR Stone, Urinary Tract[tiab] OR Stone, Urine[tiab] OR Urinary Calculi[tiab] OR Urinary Calculus[tiab] OR Urinary Stone[tiab] OR Urinary Tract Calculus[tiab] OR Urinary Tract Stone[tiab] OR Urine Calculus[tiab] OR Urine Stone[tiab] OR Urolith[tiab] OR Urolyt[tiab] OR Bladder Calculi[tiab] OR Bladder Calculosis[tiab] OR Bladder Calculus[tiab] OR Bladder Concrement[tiab] OR Bladder Concretion[tiab] OR Bladder Lithiasis[tiab] OR Bladder Neck Calculus[tiab] OR Bladder Stone Disease[tiab] OR Bladder Stones[tiab] OR Calculosis, Bladder[tiab] OR Calculus, Urinary Bladder[tiab] OR Cystolithiasis, Urinary[tiab] OR Stone, Bladder[tiab] OR Urinary Bladder Calculi[tiab] OR Urinary Bladder Stone[tiab] OR Urinary Bladder Stones[tiab] OR Vesical Calculi[tiab] OR Vesical Calculus[tiab] OR Vesical Stone[tiab] OR Vesical Stones[tiab] "Child"[mh] OR "Infant"[mh] OR "Infant, Newborn"[mh] OR "Adolescent"[mh] OR "Pediatrics"[mh] OR Children[tiab] OR Infants, Newborn[tiab] OR Newborn Infant[tiab] OR Newborn Infants[tiab] OR Newborns[tiab] OR Newborn[tiab] OR Neonate[tiab] OR Neonates[tiab] OR Adolescents[tiab] OR Adolescence[tiab] OR Teens[tiab] OR Teen[tiab] OR Teenagers[tiab] OR Teenager[tiab] OR Youth[tiab] OR Youths[tiab] #1 AND #2 AND #3

**Eligibility criteria**

We included human diagnostic value studies, which were performed to evaluate the diagnostic accuracy of ultrasonography or CT scans in identifying renal calculi in children. However, review articles, studies performing ultrasonography after CT or radiography, and duplicate articles were excluded.

**Data extraction and risk of bias**

Two independent researchers screened articles by title and abstract. Afterwards, the full-texts of potentially relevant studies were obtained and studied in detail. Next, each researcher selected eligible studies and summarized them based on a pre-designed checklist. In the event of a disagreement between the two researchers, a third reviewer studied the findings and resolved the existing disagreement by factual discussion with the other two researchers. Extracted data included relevant information about sample characteristics (age, sex), sample size, sampling method (convenience / consecutive), study design (retrospective, prospective), probe for ultrasonography, operator and interpreter of CT scan and ultrasonography findings, reference test, true positive (TP), false positive (FP), true negative (TN), false negative (FN), outcome (presence or absence of stone) and potential bias.

The risk of bias assessment of the studies was performed based on the proposed 14-item Quality Assessment of Diagnostic Accuracy Studies (QUADAS-2) tool (20).

**Statistical analysis**

Analyzes were performed using STATA 14.0 statistical program using the “*midas*” module. TP, FP, TN and FN were recorded in patients with renal kidney in each study. These numbers were used to calculate the area under the curve (AUC), sensitivity, specificity, diagnostic score and diagnostic odds ratio with 95% confidence interval (95% CI). Furthermore, a mixed-effects binary regression model, which is a kind of random effect model, was used to perform the analyses. Heterogeneity assessment was evaluated via I^2^ tests (21). Deek's funnel plot was also used to check publication bias. Finally, a subgroup analysis was performed using a bivariate mixed-effects binary regression model to identify the source of heterogeneity.

## Results:

**Study characteristics**

Initial search of databases resulted in 2512 non-redundant articles, 17 of which were reviewed in full. Finally, data from 7 studies were included in the present meta-analysis (22-28) (Figure 1). These studies included data from 1382 children with suspected renal calculi. 211 cases were TP, 1116 TN, 1 FP, and 54 FN. Except for one study, CT scan was the reference test. Only one study had reported the type of probe and its frequency in detail. Two studies stated that probe selection was based on patient status. The operator of the ultrasonography device was pediatric sonographer in one study and radiologist in 3 studies. Operator was not reported in other studies. Three studies reported ultrasonography criteria for diagnosis of renal calculi. Table 1 presents a summary of the included studies.

**Risk of bias and publication bias**

The risk of bias in patient selection was high in 5 studies. In other cases, the risk of bias was low or unclear. Also, all studies regarding the patient selection, index test and reference standard sections were applicable to the present meta-analysis (Table 2 and Figure 2). However, some degrees of publication bias were observed in the included studies (p = 0.04) (Figure 2).

**Diagnostic performance of ultrasonography**

Pooled analysis showed that the AUC of ultrasonography in diagnosis of pediatric renal calculi was 0.94 (95% CI: 0.92 to 0.96) (Figure 3). Sensitivity and specificity of this diagnostic modality were 0.80 (95% CI: 0.70 to 0.87) and 1.00 (95% CI: 0.84 to 1.00), respectively (Figure 4). Diagnostic score and diagnostic odds ratio of ultrasonography in renal calculi detection were 110.32 (95% CI: 2.88 to 19.76) and 82362.41 (95% CI: 17.80 to 3.8 × 10^8^), respectively (Figure 5).

Subgroup analysis showed that ultrasonography operator (p = 0.14), reporting ultrasonography criteria (p = 0.09) and study performance year (p = 0.33) did not have any effect on the sensitivity of ultrasonography but had a slight effect on its specificity (P <0.0001) (Table 3).

## Discussion

The present meta-analysis was the first to quantitatively summarize the findings reported in the literature on the value of ultrasonography in identifying renal calculi in children. The findings of this study showed that ultrasonography had 80% sensitivity and 100% specificity in identifying renal calculi in children. In other words, ultrasonography is an excellent diagnostic test for renal calculi, and only one case of false positive has been reported. However, it is not a perfect screening tool because its sensitivity is 80% and 54 false negatives have been observed.

In a systematic review Tasian and Copelovitch stated that ultrasound should be used as the first diagnostic modality for screening of renal calculi in children and CT scan should be used only when ultrasonography findings are negative and the suspicion of nephrolithiasis remains high (17). The conclusion reported by Tasian and Copelovitch on the use of ultrasound as the first diagnostic modality is inconsistent with the findings of the present study, because pooled sensitivity of ultrasonography in the present meta-analysis is 80%. Although Tasian and Copelovitch stated in their study that patients with high suspicion to nephrolithiasis and negative ultrasonography should undergo a CT scan, this is not clinically feasible because the patient certainly had at least one important clinical sign that led the physician to suspect renal calculi. According to this recommendation, virtually all patients with non-diagnostic ultrasonography should undergo a CT scan. Contrary to the Tasian and Copelovitch study, we do not recommend the use of ultrasonography as the first modality. In another systematic review, Hoppe and Kemper referred to ultrasonography as a modality with more advantages than other imaging tools in identifying renal calculi in children, including avoiding exposure to ionizing radiation, easy detection of hydronephrosis, and identifying some anatomical aspects of the urinary tract. However, they stated that ultrasonography is not as sensitive as CT scan in identifying small stones and the skill of the operator plays an important role in its diagnostic value (15). The findings of this study are in line with the present study.

Subgroup analysis showed that differences in ultrasonography, reporting of ultrasonography criteria for renal stone diagnosis and study performance year did not affect sensitivity. Although the effect of these factors on specificity was statistically significant, it was clinically insignificant as the specificity of ultrasonography changed by only 1%.

The use of a suitable probe is essential in increasing the sensitivity and specificity of ultrasonography (29). However, only one of the articles included in the present study provided the details of the probe used. Although the probability of using an inappropriate transducer is low, failure to report this issue precludes reaching conclusions about the role of probe type in performance of ultrasonography for pediatric renal calculi diagnosis.

Operator dependence of ultrasonography is a well-established fact (30-33). However, 3 studies did not report ultrasonography operator. This is a serious limitation as it cannot be ascertained whether the ultrasound operator is sufficiently skilled in the research performed or not. Although a subgroup analysis based on ultrasonography operator was done in the present meta-analysis, comparisons were made between studies that reported operator expertise and those that didn’t. In other words, since the expertise of the operator is unclear in some studies, the analysis of this section cannot be reliable.

Studies have used various criteria to identify renal calculi, including acoustic shadowing, echogenic focus and twinkling artifact. However, 3 of the 7 studies did not specify which ultrasonic markers they used to identify renal calculi. In addition, it should be noted that it is difficult to detect calculi in collapsed ureter using ultrasonography (29). In these cases, the patient should be well hydrated and the bladder acts as a sonic window to identify ureteral stones. Therefore, in emergency situations, when the patient is dehydrated and there is insufficient time to hydrate the patient, ultrasonography will be of limited use in identifying urinary tract calculi.

Another limitation of the included studies was the retrospective design in 5 studies. The retrospective design may increase the risk of bias in the reported findings. In retrospective studies, both the findings of ultrasonography and CT scan are available. Therefore, ultrasonography findings may be interpreted with knowledge of the CT scan findings. This cannot be completely eliminated from retrospective studies. In addition, in many cases controlling for confounding factors such as the interval between ultrasound and CT scans, the type of probe and the operator of the ultrasonography device is not possible. Therefore, the findings reported in these studies appear to be of low level of evidence.

## Conclusion:

In conclusion, the low level of evidence indicates that sensitivity and specificity of ultrasonography for detecting renal calculi in children are 80% and 100%, respectively. However, due to the serious limitations of the included studies, well-designed prospective diagnostic accuracy studies are recommended for future studies. 
